# Integrated Care: A Collaborative ADVANTAGE for Frailty

**DOI:** 10.5334/ijic.4156

**Published:** 2018-04-18

**Authors:** Anne Hendry, Ana Maria Carriazo, Eliane Vanhecke, Ángel Rodríguez-Laso, on behalf of the ADVANTAGE JA Work Package 7

**Affiliations:** 1 NHS Lanarkshire, Scotland, GB; 2 Regional Ministry of Health, Andalucía, ES; 3 Ministry of Health and Social Affairs, FR; 4 Biomedical Research Foundation of the University Hospital of Getafe, Madrid, ES

**Keywords:** integration, frailty, CGA, enablement

## Abstract

Frailty is increasingly recognised as a public health priority due to the associated demand for acute and longer term health and social care support, and the impact on the lives of individuals, caregivers and families. Integrated care is widely considered to be most effective when applied to an older population, but there is limited data on outcomes and costs from studies of integrated care to prevent and manage frailty. This paper describes work by the ADVANTAGE Joint Action (JA), co-funded by the European Union and 22 Member States, to develop a common European approach to the prevention and management of frailty. The authors reflect on the emerging evidence and experience of implementing integrated care for frailty, and invite readers to participate in ongoing dialogue on this topic through the ADVANTAGE JA website and IFIC Academy activities.

## Frailty Matters

Multimorbidity, disability and frailty are distinct clinical entities that are related, often associated, and may overlap [1]. Frailty, affecting around 11% of people over 65 years in the community [2], is more strongly predictive of disability, hospitalisation, long term care or death than is multimorbidity [3,4]. The average additional costs associated with frailty when controlled for ageing and multimorbidity range from 1,500 to 5,000 €/person per annum depending on the care setting studied [5,6,7]. However, frailty is not an inevitable consequence of ageing and emerging research is focused on earlier interventions for at risk individuals to prevent and delay the onset of frailty [8].

Frailty shares many features of a chronic condition: a dynamic syndrome that cannot be cured but may be prevented and better managed in primary care through an interdisciplinary chronic disease management approach that anticipates and proactively manages episodes of deteriorating function [9]. Integrated care has emerged as an effective way to improve outcomes for people living with chronic and complex physical and mental health conditions. However few integrated care programmes have been specifically designed to prevent and manage frailty. As a complex and multidimensional syndrome of increased vulnerability, frailty requires a well coordinated response by many different professionals and providers from health care, social care, housing, independent and community sectors.

## European Joint Action

A Joint Action (JA) is a grant for actions co-financed with Member States, or other countries participating in the Programme and the European Commission, to allow nominated authorities to take forward work on jointly identified issues that have a clear added value for the European Union under the third Health Programme 2014–2020. For ADVANTAGE JA partners, the shared issue is a belief that we must urgently review our policies on ageing and design more integrated models of health and social care to better meet the complex and changing needs of the growing number of adults at risk of, or living with, frailty.

We conducted a systematic search of peer-reviewed medical literature published from 2002 to 2017, combining the concepts of frailty and models of care. This search yielded 1065 potential articles. Articles on a specific disease, process or intervention without considering the approach to service delivery were excluded leaving 163 abstracts and 43 full papers for analysis. We also reviewed information on frailty projects funded by the European Union or registered with the European Innovation Partnership on Active and Healthy Ageing, and grey literature (including good practices) identified by ADVANTAGE partners.

A synthesis of 18 comprehensive programmes encompassing various components of the Chronic Care Model for people with multimorbidity or frailty reported some evidence of improved health-related quality of life, function, and satisfaction with care but no reduction in health services utilisation or costs [10]. These programmes included standard chronic care interventions such as case managers, multi-professional teams, and individualised care plans delivered at specific touchpoints in the care pathway. However few of the programmes were designed to prevent and tackle frailty across the full pathway of care – from community to hospital and at the interface between primary care, social care and hospital care.

## Chronic care + enablement

An important distinction between chronic disease and frailty is that frailty is more often associated with functional impairments and physical inactivity that require a restorative or enabling approach beyond the scope of a traditional chronic care model. A restorative approach to care for frail older people living at home has significant advantages over the traditional model of home care maintenance and support [11]. Timely interventions, education and assistive technologies specifically designed to encourage frail older people to resume activity and regain independence may be cost-effective by reducing disability and future demand for services [12]. Therefore we consider that a comprehensive frailty prevention approach should adopt a behavioural health, education and enablement ethos and include interventions such as a home exercise programme, support to regain skills such as cooking or dressing, and to build social networks that reduce isolation, depression and anxiety.

## CGA + care transitions

Comprehensive assessment, individualised care plans, and coordination of tailored interventions are the essence of Comprehensive Geriatric Assessment (CGA): a highly evidence based approach that improves care for frail older people in hospital and increases the likelihood of patients living in their own homes at three to 12 months after discharge [13]. There has been more limited study of the CGA approach in community settings and at times of transition between home and hospital. Two overviews of community programmes illustrate components that mirror the CGA approach [14,15] but few primary care or community studies included access to urgent community assessment and advice after office hours or at weekends. We suggest that optimal care for frailty and system benefits will not be realised until well coordinated health and social care assessment and support can be accessed across care settings and at all times. We consider this can be facilitated by remote monitoring and decision support in the community and by urgent access to intermediate care or transition services such as Hospital at Home [16,17].

## Scaling up the gains

Varying results of community based studies of integrated care for frailty may be due to different sample size, casemix, fidelity, staff expertise, access to care out of hours and duration of study and follow up. Most were small scale demonstration projects that have yet to spread across larger geographic areas, or population groups. In our experience, large scale integrated care models require a favourable political and funding context that may only be achieved through changes in legislation, policy, funding and delivery arrangements as we have recently observed in France, Spain, and Scotland [18,19].

The evidence of cost effectiveness from well executed economic evaluations of models of care is limited. Economic benefits of implementing system-level changes at scale are well described in the Program of Research to Integrate the Services for the Maintenance of Autonomy (PRISMA) in Quebec [20]. However it is noticeable that the positive results were not reached until year three when implementation approached 80% and physician participation was 73%. Successful implementation at scale therefore demands tenacity from policy makers, managers, professionals and funders.

We believe that more large-scale studies with longer intervention and follow up periods are needed to evaluate system outcomes and costs of integrated care to prevent and manage frailty. Moreover, studies to date have generally focused on traditional healthcare outcomes. Our vision is that future models of care should be designed around outcomes that matter for individuals and their caregivers as well as health and care system and societal impact. We suggest that a focus on patient, client or user-defined goals and outcomes should capture care experience, quality of life and participation outcomes in addition to function and traditional health and social care metrics.

## Emerging lessons

Current evidence supports the case for a holistic response to frailty that blends a chronic care approach with education, enablement and rehabilitation to optimise function, particularly at times of a sudden change in health status, or when moving between home, hospital or care home. In all care settings, these approaches should be supported by comprehensive assessment and multidimensional interventions tailored to modifiable physical, psychological, cognitive and social factors and appropriate to the goals and circumstances of the individual and their caregiver.

We suggest that model of care for frailty should incorporate the following components:

- a single entry point in the community – generally in Primary Care
- use of simple frailty specific screening tools in all care settings
- comprehensive assessment and individualised care plans – including for caregivers
- tailored interventions by an interdisciplinary team – both in hospitals and community
- case management and coordination of support across the continuum of providers
- effective management of transitions between care teams and settings
- shared electronic information tools and technology enabled care solutions
- clear policies and procedures for service eligibility and care processes.

## Join the conversation

More details on our review of integrated care for frailty are available at the JA website www.advantageja.eu along with a *State of the Art on Frailty Report* that has information on frailty definition and diagnosis; epidemiology, population screening, monitoring and surveillance; prevention, clinical management; education/training of the workforce; and research. ADVANTAGE JA partners will facilitate a series of sessions on frailty at ICIC18, Utrecht 23–25^th^ May, under *Theme 5: Vulnerable populations and populations at risk*.

The aim will be to:

- raise awareness of Frailty as a high impact priority for integrated care
- highlight examples of good practice in preventing and managing frailty
- extend our ADVANTAGE community of practice
- establish an IFIC Academy special interest group for frailty

For further information on ADVANTAGE JA and IFIC special interest group please contact the authors at the address below Anne.hendry@lanarkshire.scot.nhs.uk.

## References

[1] Cesari, M, Pérez-Zepeda, MU and Marzetti, E. Frailty and Multimorbidity: Different Ways of Thinking About Geriatrics. Journal of the American Medical Directors Association, 2017; 18(4): 361–364. DOI: 10.1016/j.jamda.2016.12.086 28279606

[2] Collard, RM, Boter, H, Schoevers, RA and Oude Voshaar, RC. Prevalence of frailty in community-dwelling older persons: A systematic review. J Am Geriatr Soc, 2012; 60: 1487–1492. DOI: 10.1111/j.1532-5415.2012.04054.x 22881367

[3] Kan, GA, Rolland, Y, Bergman, H, Morley, JE, Kritchevsky, SB and Vellas, B. The I.A.N.A. task force on frailty assessment of older people in clinical practice. The Journal of Nutrition Health and Aging, 2008; 12(1): 29–37. DOI: 10.1007/BF02982161 PMC12892609 18165842

[4] Gill, TM. Transitions between frailty states among community-living older persons. Archives of Internal Medicine, 2006; 166(4): 418–423. DOI: 10.1001/archinte.166.4.418 16505261

[5] Bock, J, König, H, Brenner, H, Haefeld, WE, Quinzler, R, Matschinger, H, Saum, K, Schöttker, B and Heider, D. Associations of frailty with health care costs – results of the ESTHER cohort study. BMC Health Services Research, 2016; 16: 128 DOI: 10.1186/s12913-016-1360-3 27074800 PMC4831082

[6] Sirven, N and Rapp, T. The cost of frailty in France. The European Journal of Health Economics, 2016; 18(2): 243–253. DOI: 10.1007/s10198-016-0772-7 26914932

[7] García-Nogueras, I, Aranda-Reneo, I, Peña-Longobardo, LM, Oliva-Moreno, J and Abizanda, P. Use of health resources and healthcare costs associated with frailty: The FRADEA study. The journal of nutrition, health & aging, 2016; 21(2): 207–214. DOI: 10.1007/s12603-016-0727-9 PMC12879863 28112778

[8] Mohandas, A, Reifsnyder, J, Jacobs, M, and Fox, T. Current and Future Directions in Frailty Research. Population Health Management, 2011; 14(6): 277–283. DOI: 10.1089/pop.2010.0066 22087470

[9] Harrison, JK, Clegg, A, Conroy, S and Young, J. Managing frailty as a long-term condition. Age and Ageing, 2015; 44: 732–735 DOI: 10.1093/ageing/afv085 26175349

[10] Hopman, P, de Bruin, SR, Forjaz, MJ, Rodriguez-Blazquez, C, Tonnara, G, Lemmens, LC, Onder, G, Baan, C and Rijken, M. Effectiveness of comprehensive care programs for patients with multiple chronic conditions or frailty: A systematic literature review. Health Policy, 2016; 120: 818–823. DOI: 10.1016/j.healthpol.2016.04.002 27114104

[11] Ryburn, B, Wells, Y and Foreman, P. Enabling independence: restorative approaches to home care provision for frail older adults. Health and Social Care in the Community, 2009; 17(3): 225–234. DOI: 10.1111/j.1365-2524.2008.00809.x 19175428

[12] Markle-Reid, M, Browne, G and Gafin, A. Nurse led health promotion interventions improve quality of life in frail older home care clients: lessons learnt from three randomised trials in Ontario, Canada. J Eval Clin Pract, 2013; 19(1): 118–131. DOI: 10.1111/j.1365-2753.2011.01782.x 22029487

[13] Ellis, G, Gardner, M, Tsiachristas, A, Langhorne, P, Burke, O, Harwood, RH, Conroy, SP, Kircher, T, Somme, D, Saltvedt, I, Wald, H, O’Neill, D, Robinson, D and Shepperd, S. Comprehensive geriatric assessment for older adults admitted to hospital. Cochrane Database of Systematic Reviews, 2017; Issue 9 Art. No.: CD006211 DOI: 10.1002/14651858.CD006211.pub3 28898390 PMC6484374

[14] Béland, F and Hollander, MJ. Integrated models of care delivery for the frail elderly: international perspectives. Gac Sanit., 2011 12; 25 Suppl 2: 138–46. DOI: 10.1016/j.gaceta.2011.09.003 22088903

[15] Threapleton, DE, Chung, RY, Wong, SYS, Wong, E, Chau, P, Woo, J, Chung, VCH and Yeoh, E. Integrated care for older populations and its implementation facilitators and barriers: A rapid scoping review. International Journal for Quality in Health Care, 2017; 29(3): 327–334. DOI: 10.1093/intqhc/mzx041 28430963

[16] Mas, M, Inzitari, M, Sabate, S, Santaguenia, SJ and Miralles, R. Hospital-at-home Integrated Care Programme for the management of disabling health crises in older patients: comparison with bed-based Intermediate Care. Age and Ageing, 2017; 0: 1–7. DOI: 10.1093/ageing/afx099 28655169

[17] Shepperd, S, Doll, H, Angus, RM, Clarke, MJ, Iliffe, S, Kalra, L, Ricauda, NA, Tibaldi, V and Wilson, AD. Avoiding hospital admission through provision of hospital care at home: a systematic review and meta-analysis of individual patient data. CMAJ, 2009; 180(2): 175–82. DOI: 10.1503/cmaj.081491 19153394 PMC2621299

[18] Hendry, A. Creating an enabling political environment that supports health and social care integration. International Journal of Integrated Care, 2016; 16(4): 7, pp. 1–3, DOI: 10.5334/ijic.2531 PMC5354203 28316547

[19] Hendry, A, Taylor, A, Mercer, S and Knight, P. Improving outcomes through transformational health and social care integration: the Scottish Experience. Healthcare Quarterly, 7 2016; 19(2): 73–79. DOI: 10.12927/hcq.2016.24703 27700978

[20] MacAdam, M. PRISMA: Program of Research to Integrate the Services for the Maintenance of Autonomy. A system-level integration model in Quebec. Int J Integr Care, Special Issue: Integrating Care to Older People and those with Complex Needs. 2015; DOI: 10.5334/ijic.2246 PMC4583074 26417212

